# Continuum of care in maternal, newborn and child health in Pakistan: analysis of trends and determinants from 2006 to 2012

**DOI:** 10.1186/s12913-017-2111-9

**Published:** 2017-03-09

**Authors:** Sarosh Iqbal, Sidra Maqsood, Rubeena Zakar, Muhammad Zakria Zakar, Florian Fischer

**Affiliations:** 1 Institute of Social and Cultural Studies, University of the Punjab, Lahore, Pakistan; 2 Department of Public Health Medicine, School of Public Health, Bielefeld University, Bielefeld, Germany

**Keywords:** Pakistan, MNCH, Continuum of care, Skilled birth attendants, ANC, PNC

## Abstract

**Background:**

Pakistan, being a developing country, presents the dismal picture of maternal and neonatal mortality and morbidity. The majority of maternal and neonatal deaths could be avoided if Continuum of Care (CoC) is provided in a structured pathway from pregnancy to birth and to the first week of life of the newborn child. This study aimed to analyse the trends of CoC at all three levels (antenatal care, skilled delivery and postpartum care) and to identify various factors affecting the continuation in receiving CoC in Pakistan during 2006 to 2012.

**Methods:**

Secondary data analysis was performed on nationally representative data from the last two iterations of Pakistan Demographic and Health Survey (PDHS), conducted during 2006/07 to 2012/13. The analysis is limited to women of the reproductive age group (15–49 years) who gave birth during the last five years preceding both surveys. This leads to a sample size of 5,724 and 7,461 respondents from PDHS 2006/07 and 2012/13 respectively. The association between CoC and several factors, including individual attributes (reproductive status), family influences, community context, as well as cultural and social values was assessed in bivariate analyses in a first step. Furthermore, odds ratios and adjusted odds ratios with 95% confidence intervals using a binary and multivariable logistic regression were calculated.

**Results:**

Our research presents the trends of a composite measure of CoC including antenatal care, delivery assistance and postpartum care. The largest gap in CoC was observed at antenatal care followed by delivery and postnatal care within 48 h after delivery. Results show that CoC completion rate has increased from 15% to 27% amongst women in Pakistan over time from 2006 to 2012. Women with high age at first birth, having less number of children, with higher education, belonging to richest quintile, living in Sindh province and urban areas, having high autonomy and exposure to mass media were most likely to avail complete CoC.

**Conclusions:**

The findings show that women in Pakistan still lack the CoC. This calls for attention to develop and implement tailored interventions, focusing on the needs of women in Pakistan to provide CoC in an integrated manner, involving both public and private sectors by appropriately addressing the factors hindering CoC completion rates.

## Background

Reducing the global burden of preventable maternal, newborn and child mortality and morbidity is a key focus for public health. Accelerating progress earlier towards Millennium Development Goals (MDGs) and now Sustainable Development Goals (SDGs) for improving the maternal, newborn and child health (MNCH) has attained the top priority within the global political agenda [1, 2]. World Health Organization (WHO) recent estimates highlight that approximately 303,000 maternal deaths occurred globally in 2015. It indicates that on average 830 maternal deaths happen each day, with a majority of deaths in developing countries. Most of the deaths occur during labour, delivery and the immediate postpartum period and could be avoided. Similarly is the case of neonatal and child mortality, with globally 5.9 million deaths in children under five years in 2015, including 2.7 million newborns within the first 28 days after birth (equivalent to 45% deaths of children under five years) [3].

Under a global perspective, it is estimated that approximately 80% of maternal deaths and up to two thirds of neonatal deaths could be avoided if effective health measures are provided during birth and first week of life [4, 5]. Considering the notion that most of the maternal and child deaths are preventable, the burden of mortality and morbidity is unacceptably high. A simple, cost-effective and low-technology intervention, e.g. Continuum of Care (CoC) to cope MNCH challenges, is required. The CoC approach denotes to the continuation of care throughout the lifecycle including adolescence, pregnancy, childbirth, postnatal period and childhood for improving the health and survival of mothers and children [6]. In the past, MNCH tended to address the mother and child separately. This resulted in gaps within care affecting newborn babies in particular. However, the concept of CoC implies that MNCH is closely connected and must be dealt in an integrated manner. The CoC emphasizes on two key dimensions, i.e. time and place. The time dimension highlights the continuity of care over time at different stages of pregnancy, childbirth and postpartum. The place dimension links various levels of services provided at home, communities and health facilities [6–9].

Considering the scenario mentioned above, we have applied a narrowed scope of CoC for our research, paying attention to the time dimension for continuity of care at each of the three maternal health services during the period from pregnancy over childbirth to the time after delivery. The featured services included antenatal care (ANC) during pregnancy, skilled birth attendance (SBA) during delivery and postnatal care (PNC) for mothers and newborn during postpartum period. Firstly, all pregnant women should have adequate and high-quality ANC during pregnancy, at least by attaining the optimum number of four visits as advocated by WHO [10]. Timely visits of ANC and its related contents help women for birth preparedness, enabling them to identify and treat illness during pregnancy, as well as to use health facilities for emergency obstetric care [11]. Secondly, women should have skilled birth attendance from qualified and experienced professionals (e.g. doctors, nurses, midwifes, lady health visitors [LHV]) during childbirth for safe and normal delivery, who are well-equipped with drugs and supplies necessary for effective prevention, management and also for referral in case of any obstetric complications [12]. Lastly, women should have continued care after delivery for themselves and also for their newborns, as the postpartum time is a crucial phase to avoid complications, which could result in maternal or newborn mortality. Overall, completion of the continuum of care follows a pathway from pregnancy to delivery to postpartum, where each step adds a value to ensure better health outcomes for mothers and newborns, and also contributes in maternal and neonatal mortality reduction [13].

### Aims and objectives

Despite substantial progresses in improving MNCH indicators during the last decade in Pakistan, more efforts are still required to save the lives of mothers and children. Extensive literature focussing on the context of Pakistan is available, which explores the various influencing factors regarding the use of individual maternal health services, particularly for ANC and SBA. Nevertheless, this research is unique in nature as it focuses on varied pattern on utilization of CoC during the last few years. The main objectives of this research are to analyse the trends of CoC amongst women of reproductive age along the pathway from pregnancy to childbirth to postpartum period during 2006 to 2012 and to identify various factors affecting continuation in receiving CoC in Pakistan.

## Methods

### Data source

The analysis is based on data from the last two iterations of Pakistan Demographic and Health Survey (PDHS), conducted during 2006/07 (wave 2) and 2012/13 (wave 3). The PDHS is a nationally representative, large-scale and repeated cross-sectional survey, produced by ORC Macro for the Measure DHS (Demographic and Health Surveys) Project and funded by US Agency for International Development (USAID). These surveys used a multistage cluster sampling design to collect data on reproductive health, fertility, mortality, family planning, nutrition and health care utilization. The information on selected maternal and child health indicators were taken from the woman’s questionnaire. The data was collected from women aged 15–49 years administering a standard questionnaire. Information are based on self-reports. Due to the same sampling design, estimates obtained from the two waves of PDHS are comparable. The details of surveys design, methodology, data collection and management procedures are described in the reports of the respective waves of PDHS [14, 15].

### Study population

The overall sample sizes were 10,023 and 13,558 ever-married women from PDHS 2006/07 and PDHS 2012/13 respectively. The population for this research included women of reproductive age group (15–49 years) who gave birth during the last five years preceding both surveys, which yielded to sample sizes of 5,724 and 7,461 respondents from PDHS 2006/07 and PDHS 2012/13. The reason for selecting women giving birth during the past five years preceding the survey was to avoid memory recall bias of the mother. All participants provided written informed consent to participate.

### Outcome variables

Complete continuum of care for MNCH services is the outcome variable of research. The composite CoC was constructed into a binary variable. For that purpose, CoC was classified as *complete* when the women reported that they received services at the following three levels, either at health facility or at home:

- At least four ANC visits (ANC4+) conducted by respondents during pregnancy for healthcare check-ups;
- Deliveries assisted by a qualified skilled health professional, e.g. doctor, nurse, midwife or LHV; and
- PNC check-ups of mothers and newborns within 41 days or six weeks after childbirth.

CoC was classified as *discontinued*, if the mother skipped any one of these steps. Furthermore, achievement of ANC4+ visits was defined as continued care at pregnancy level while achievement of SBA with ANC4+ was considered as continued care at delivery level. Ultimately, achievement of ANC4+, SBA and PNC was measured as continued care at postpartum level, which is also interpreted as complete CoC.

Because neither ANC4+ visits nor further ante- or postnatal services are recorded by health facilities in Pakistan [16], the information is based on self-reports. According to the definition by Utz et al. [17], LHV are considered as skilled health professionals, although the length of their training is not equivalent to other disciplines such as doctors, nurses or midwifes.

The internal consistency of these composite CoC variables was tested. Cronbach’s alpha reliability test showed significant results for both waves of the survey (PDHS 2006/07: 0.47; PDHS 2012/13: 0.58).

### Independent variables

**Fig. 1:**
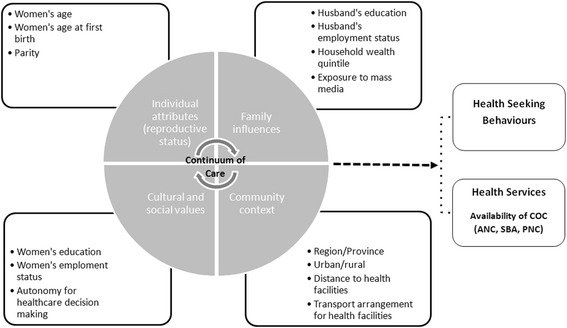
Conceptual framework of research

Based on a literature review regarding various factors associated with CoC, we adapted a conceptual framework which was originally developed to analyse maternal mortality and the social dimensions of maternal health (Fig. 1) [18–20]. This framework illustrates several determinants influencing the maternal healthcare utilization. Individual attributes of reproductive status, family influences, community context, as well as cultural and social values are included in this framework.

The determinants related to individual attributes of the reproductive status include respondents age, age at first birth and parity. Information on family influences was assessed through husbands educational and employment status, composite index of household amenities (wealth quintiles) and exposure to mass media (which refers to the frequency of reading a newspaper, watching TV or listening to radio to access relevant information). The determinants of the community context comprise of respondents provincial/regional location, type of geographical classification into urban and rural areas, their distance to health facilities and transport arrangements for health facilities to receive medical care. Here, the distance to a facility and transport arrangement refers to the respondent issue of accessibility which prevent them from receiving a medical advice or treatment, in case when she is sick. Under determinants of cultural and social values, respondents’ education, employment status and their autonomy for healthcare decision making are included. This information was assessed by posing the following question: “Who usually makes decisions about health care for yourself: you, your husband/partner, you and your husband/partner jointly, or someone else?”

### Statistical analysis

Data was analysed using SPSS version 21. Descriptive statistics were used to report findings of CoC at three levels through frequencies and percentages. Simple bivariate and multivariate logistic regression analyses were applied for modelling to identify the association among various factors at all three levels of CoC. Odds ratios (OR) and adjusted odds ratios (AOR) with 95% confidence intervals (CI) and p-values were calculated. The significance level was assigned at *p* ≤ 0.05.

## Results

### Socio-demographic characteristics of respondents

Table 1 indicates the socio-demographic characteristics of the respondents (women of reproductive age 15–49 years) who gave birth during the last 5 years within each of the two waves of PDHS during 2006 to 2012. Most of the women were from rural areas (65 and 56%) and amongst the age group of 25–34 years (51 and 53%), from wave 2 and 3 respectively. The majority gave first birth at the age of 20–29 years. In both waves, the majority of respondents were uneducated (67 and 55%) and unemployed (72 and 79%). Contrary to respondents, most of the husbands were found employed (97% for both waves).

**Table 1:** Socio-demographic characteristics of respondents of reproductive age (15-49 years) who gave birth during the last five years within each of the two waves of PDHS during 2006-2012

Characteristics	PDHS (2006/07)		PDHS (2012/13)	
	*n*=5,724		*n*=7,461	
	*n*	%	*n*	%
Regions/Provinces				
Punjab	2,305	40.3	2,008	27.0
Sindh	1,626	28.4	1,591	21.3
Baluchistan	1,113	19.4	1,149	15.4
Khyber Pakhtunkhwa	680	11.9	1,532	20.5
Gilgit Baltistan	-	-	709	9.5
Islamabad	-	-	472	6.3
Geographical classification				
Urban	1,998	34.9	3,278	43.9
Rural	3,726	65.1	4,183	56.1
Respondents age				
15-24 years	1,377	24.1	1,710	22.9
25-34 years	2,925	51.1	3,967	53.2
35 years and above	1,422	24.8	1,784	23.9
Respondents educational status				
Uneducated	3,811	66.6	4,121	55.2
Primary	789	13.8	1,065	14.3
Secondary	763	13.3	1,373	18.4
Higher	361	6.3	902	12.1
Husbands educational status				
Uneducated	2,088	36.6	2,293	30.8
Primary	921	16.1	1,014	13.6
Secondary	1,788	31.4	2,441	32.8
Higher	906	15.9	1,689	22.7
Respondents employment status				
Unemployed	4,092	71.5	5,936	79.6
Employed	1,628	28.5	1,524	20.4
Husbands employment status				
Unemployed	191	3.3	197	2.6
Employed	5,531	96.6	7,262	97.4
Respondents age at first birth				
Less than 20 years	2,549	44.5	2,952	39.6
20-29 years	2,985	52.1	4,243	56.9
30 years and above	190	3.3	266	3.6
Parity/Number of children				
1-2 children	2,024	35.4	2,828	37.9
3-4 children	1,627	28.4	2,249	30.1
5 children or more	2,073	36.2	2,384	32.0
Wealth quintile				
Richest	1,289	22.5	1,503	20.1
Richer	1,235	21.6	1,423	19.1
Middle	1,118	19.5	1,429	19.2
Poorer	1,064	18.6	1,483	19.9
Poorest	1,018	17.8	1,623	21.8
Exposure to mass media^a^				
Yes	-	-	5,204	70.0
No	-	-	2,226	30.0
Respondents autonomy in healthcare decision-making				
Yes	-	-	3,221	43.7
No	-	-	4,146	56.3
Distance to health facility for medical care				
Big Problem	-	-	3,237	43.5
Not a big problem	-	-	4,209	56.5
Transport arrangement for medical care				
Big Problem	-	-	3,555	47.7
Not a big problem	-	-	3,891	52.3

^a^refers to the frequency of reading a newspaper or watching TV or listening to radio

Four parameters were only analysed during the last wave of PDHS (2012/13): exposure to mass media, autonomy and accessibility to health facility for medical care, with respect to problem of distance and transport arrangements. Findings highlight that around 70% of women had access to mass media, 44% had high autonomy in healthcare decision-making, whereas the majority of women considered that distance to a health facility (56.5%) and the transport arrangement to reach a facility (52.3%) are not big problems to seek medical care.

### Changing trends on measures of continuum of care

Table 2 shows the measures used to describe CoC among respondents of reproductive age within the two waves of PDHS during 2006 to 2012. Findings revealed that only 28 and 38% of respondents had availed ANC4+ during waves 2 and 3 respectively, showing a positive change with 10 percentage points increase in achievement of ANC4+ over a period of time.

**Table 2:** Measures to describe Continuum of Care for MNCH services within each of the two waves of PDHS during 2006-2012

Characteristics	PDHS (2006/07)		PDHS (2012/13)		PDHS (2006/07)	PDHS (2012/13)	Gap over time
	*n*=5,724		*n*=7,461		mean		
	*n*	%	*n*	%	%		
Continuum of Care for MNCH							
Pregnancy level: Antenatal care - at least four visits﻿ (ANC4+)							
At least 4 visits or more	1,559	27.7	2,859	38.4	28	38	10
Less than 4 visits	4,077	72.3	4,584	61.6			
Delivery level: Skilled birth attendan﻿ce at delivery (SBA)							
Yes	2,408	42.1	4,224	56.8	42	57	15
No	3,292	57.5	3,216	43.2			
Postpartum level: Postnatal care (PNC) for mothers and newborn							
Yes	2,755	48.5	4,708	63.2	48	63	15
No	2,927	51.5	2,738	36.8			
Continuum of Care (CoC)							
Continued Care at pregnancy							
Yes, received ANC4+	1,559	27.7	2,859	38.4	28	38	10
Not received	4,077	72.3	4,584	61.6			
Continued Care at delivery							
Yes, received ANC4+ and SBA	1,139	20	2,397	32.3	20	32	12
Not received	4,565	80	5,033	67.7			
Complete Continuum of Care^a^							
Continued Care at all three levels (ANC4+, SBA and PNC)	852	14.9	2,045	27.4	15	27	12
Discontinued Care	4,852	85.1	5,416	72.6			

^a^This may also interpreted as Continued Care at Postpartum

Regarding SBA and PNC for mothers and newborns, an increase of 15 percentage points was detected during the two PDHS waves. The same trend was observed amongst women in following the pathway of complete CoC, where 15% respondents in wave 2 and 27% in wave 3 attained continued care at all three levels.

### Associations of achievement of continued care at pregnancy, delivery and postpartum (complete CoC) with various factors

Tables 3–6 indicate the bivariate and multivariate analyses regarding the achievement of ANC4+ (continued care during pregnancy), achievement of ANC4+ and SBA (continued care during delivery) and achievement of ANC4+, SBA and PNC (complete continuum of care) with various determinants of respondents who gave birth in the last five years before the two waves of PDHS from 2006 to 2012.

**Table 3:** Association of three levels of Continuum of care (CoC) with various key determinants within each of the two waves of PDHS during 2006-2012

Characteristics	PDHS (2006/07)									PDHS (2012/13)								
	*n*=5,724									*n*=7,461								
	Care at pregnancy			Care at delivery			Complete Continued Care			Care at pregnancy			Care at delivery			Complete Continued Care		
	Cont. Care	Discont. Care	*p*-value^a^	Cont. Care	Discont. Care	*p*-value^a^	Cont. Care	Discont. Care	*p*-value^a^	Cont. Care	Discont. Care	*p*-value^a^	Cont. Care	Discont. Care	*p*-value^a^	Cont. Care	Discont. Care	*p*-value^a^
	%			%			%			%			%			%		
Geographical classification																		
Urban	56.2	26.7	<0.01	63.2	27.9	<0.01	64.9	29.7	<0.01	61.3	33.1	<0.01	64.8	34.1	<0.01	65.2	35.9	<0.01
Rural	43.8	73.3		36.8	72.1		35.1	70.3		38.7	66.9		35.2	65.9		34.8	64.1	
Respondents age																		
15-24 years	23.3	24.5	<0.01	23.4	24.3	<0.01	22.8	24.4	<0.01	23.5	22.6	<0.01	23.6	22.6	<0.01	23.7	22.6	<0.01
25-34 years	57.2	49		58.7	49.2		60.8	49.4		56.3	51.2		56.6	51.6		56.1	52.0	
35 years and above	19.4	26.5		17.8	26.5		16.4	26.3		20.2	26.2		19.8	25.8		20.1	25.3	
Respondents age at first birth																		
Less than 20 years	37.7	47.3	<0.01	32.4	47.6	<0.01	29.8	47.2	<0.01	29.6	45.8	<0.01	27.5	45.3	<0.01	25.3	44.9	<0.01
20-29 years	58.6	49.6		63.2	49.3		66.0	49.7		64.7	51.9		66.0	52.5		67.7	52.8	
30 years and above	3.7	3.2		4.4	3.1		4.2	3.2		5.7	2.3		6.4	2.2		6.9	2.3	
Respondents educational status																		
Uneducated	41.2	76.3	<0.01	33.4	74.9	<0.01	31.6	72.8	<0.01	29.3	71.4	<0.01	24.3	69.9	<0.01	22.2	67.7	<0.01
Primary	16.7	12.6		16.7	13.0		16.3	13.3		15.7	13.4		15.0	13.9		15.2	13.9	
Secondary	24.8	8.9		27.7	9.7		27.9	10.8		29.7	11.4		31.6	12.2		31.2	13.6	
Higher	17.3	2.2		22.3	2.3		24.2	3.2		25.4	3.8		29.2	4.0		31.4	4.8	
Husbands educational status																		
Uneducated	20.5	42.8	<0.01	16.1	41.8	<0.01	15.4	40.4	<0.01	15.7	40.3	<0.01	12.6	39.4	<0.01	11.5	38.1	<0.01
Primary	12.4	17.5		11.0	17.4		10.2	17.1		10.7	15.5		9.6	15.5		9.4	15.2	
Secondary	38	29		39.1	29.4		38.2	30.1		38.0	29.6		38.1	30.4		38.2	30.8	
Higher	29.1	10.7		33.7	11.4		36.1	12.3		35.6	14.6		39.6	14.8		40.9	15.9	
Respondents employment status																		
Unemployed	77.0	69.3	<0.01	80.0	69.3	<0.01	78.9	70.2	<0.01	83.3	77.2	<0.01	85.1	76.9	<0.01	84.4	77.7	<0.01
Employed	23.0	30.7		20.0	30.7		21.1	29.8		16.7	22.8		14.9	23.1		15.6	22.3	
Husbands employment status																		
Unemployed	3.0	3.5	0.34	2.9	3.5	0.34	2.2	3.5	0.05	1.9	3.1	<0.01	1.8	3.0	<0.01	1.5	3.1	<0.01
Employed	97.0	96.5		97.1	96.5		97.8	96.5		98.1	96.9		98.2	97.0		98.5	96.9	
Parity/Number of children																		
1-2 children	42.4	32.8	<0.01	47.2	32.5	<0.01	48.2	33.2	<0.01	49.6	30.7	<0.01	52.5	30.9	<0.01	53.8	31.9	<0.01
3-4 children	30.6	27.7		30.6	27.9		31.6	27.8		30.3	30.0		30.2	30.2		30.1	30.2	
5 children or more	27.0	39.5		22.2	39.7		20.2	39.0		20.1	39.3		17.3	38.9		16.1	37.9	
Respondents autonomy to healthcare decision-making																		
Yes	-	-	-	-	-	-	-	-	-	52.3	38.3	<0.01	54.1	38.9	<0.01	54.0	39.8	<0.01
No	-	-	-	-	-	-	-	-	-	47.7	61.7		45.9	61.1		46.0	60.2	
Exposure to mass media																		
Yes	-	-	-	-	-	-	-	-	-	87.4	59.1	<0.01	89.9	60.5	<0.01	91.2	62.0	<0.01
No	-	-	-	-	-	-	-	-	-	12.6	40.9		10.1	39.5		8.8	38.0	
Wealth quintile																		
Richest	40.8	9.0	<0.01	48.6	10.1	<0.01	51.4	11.9	<0.01	40.1	7.7	<0.01	44.8	8.5	<0.01	48.2	9.5	<0.01
Richer	24.8	16.1		24.8	17.0		23.7	17.6		24.5	15.6		25.0	16.2		24.6	17.0	
Middle	14.6	21.4		12.7	21.2		12.4	20.8		15.8	21.2		14.5	21.5		13.6	21.3	
Poorer	11.5	25.6		8.8	24.8		7.5	24.1		12.0	24.9		10.7	24.1		8.9	24.0	
Poorest	8.2	27.9		5.0	26.9		4.9	25.6		7.6	30.6		5.0	29.7		4.6	28.2	
Distance from health facility for medical care																		
Big problem	-		-			-	-	-	-	28.7	52.7	<0.01	25.2	52.0	<0.01	23.9	50.9	<0.01
Not a big problem	-		-			-	-	-	-	71.3	47.3		74.8	48.0		76.1	49.1	
Transport arrangement for medical care																		
Big problem	-		-			-	-	-	-	31.8	57.7	<0.01	28.3	56.9	<0.01	27.1	55.5	<0.01
Not a big problem	-		-			-	-	-	-	68.2	42.3		71.7	43.1		72.9	44.5	

^a^Chi-square test was applied to measure *p*-value

**Table 4:** Bivariate and multivariate logistic regression models of achievement of ANC4+ (Continued Care at Pregnancy) with various determinants within each of the two waves of PDHS during 2006-2012

Characteristics	Model I (ANC4+)											
	PDHS (2006/07)						PDHS (2012/13)					
	Bivariate			Multivariate			Bivariate			Multivariate		
	OR	CI (95%)	*p*-value	AOR	CI (95%)	*p*-value	OR	CI (95%)	*p*-value	AOR	CI (95%)	*p*-value
Regions/Provinces												
Baluchistan	1			1			1			1		
Punjab	4.39	3.32-5.79	<0.01	3.01	2.21-4.10	<0.01	3.94	3.27-4.74	<0.01	2.42	1.94-3.03	<0.01
Sindh	4.95	3.73-6.57	<0.01	4.96	3.63-6.77	<0.01	5.17	4.27-6.25	<0.01	4.30	3.45-5.36	<0.01
Khyber Pakhtunkhwa	2.71	2.01-3.66	<0.01	2.27	1.64-3.16	<0.01	2.37	1.95-2.88	<0.01	2.00	1.59-2.50	<0.01
Gilgit Baltistan	-	-	-	-	-	-	3.27	2.62-4.08	<0.01	3.69	2.84-4.79	<0.01
Islamabad	-	-	-	-	-	-	21.01	15.97-27.65	<0.01	6.70	4.86-9.23	<0.01
Geographical classification												
Rural	1			1			1			1		
Urban	3.52	3.12-3.97	<0.01	1.37	1.17-1.60	<0.01	3.20	2.90-3.52	<0.01	1.20	1.04-1.38	0.01
Respondents age												
15-24 years	1			1			1			1		
25-34 years	1.23	1.06-1.41	<0.01	1.17	0.96-1.43	0.12	1.06	0.94-1.18	0.35	1.04	0.88-1.24	0.62
35 years and above	0.77	0.65-0.92	<0.01	1.10	0.83-1.46	0.51	0.74	0.64-0.85	<0.01	1.02	0.79-1.31	0.86
Respondents age at first birth												
Less than 20 years	1			1			1			1		
20-29 years	1.48	1.31-1.67	<0.01	0.95	0.81-1.12	0.54	1.92	1.74-2.13	<0.01	1.18	1.04-1.36	0.01
30 years and above	1.45	1.05-2.00	0.02	1.12	0.73-1.72	0.60	3.86	2.98-5.00	<0.01	1.51	1.04-2.19	0.03
Respondents educational status												
Uneducated	1			1			1			1		
Primary	2.46	2.07-2.92	<0.01	1.37	1.13-1.67	<0.01	2.85	2.47-3.29	<0.01	1.44	1.22-1.69	<0.01
Secondary	5.13	4.34-6.06	<0.01	1.92	1.56-2.36	<0.01	6.37	5.58-7.28	<0.01	2.14	1.80-2.54	<0.01
Higher	14.46	11.22-18.62	<0.01	3.63	2.67-4.99	<0.01	16.20	13.51-19.43	<0.01	3.22	2.53-4.09	<0.01
Husbands educational status												
Uneducated	1			1			1			1		
Primary	1.48	1.21-1.81	<0.01	0.99	0.80-1.23	0.95	1.76	1.49-2.09	<0.01	1.07	0.88-1.29	0.48
Secondary	2.74	2.35-3.20	<0.01	1.36	1.13-1.64	<0.01	3.29	2.88-3.75	<0.01	1.34	1.14-1.57	<0.01
Higher	5.67	4.74-6.77	<0.01	1.53	1.21-1.93	<0.01	6.25	5.42-7.20	<0.01	1.23	1.01-1.49	0.03
Respondents employment status												
Unemployed	1			1			1			1		
Employed	0.67	0.59-0.77	<0.01	0.98	0.83-1.16	0.85	0.68	0.62-0.77	<0.01	0.92	0.78-1.07	0.27
Husbands employment status												
Unemployed	1			1			1			1		
Employed	1.18	0.84-1.65	0.34	0.94	0.64-1.39	0.77	1.67	1.22-2.29	0.01	1.22	0.84-1.77	0.30
Parity/Number of children												
5 children or more	1			1						1		
3-4 children	1.61	1.38-1.88	<0.01	0.97	0.80-1.18	0.82	1.97	1.73-2.23	<0.01	1.09	0.92-1.29	0.30
1-2 children	1.89	1.64-2.18	<0.01	1.16	0.92-1.46	0.20	3.15	2.80-3.55	<0.01	1.66	1.35-2.03	<0.01
Respondents autonomy to healthcare decision-making												
No	-	-	-	-	-	-	1			1		
Yes	-	-	-	-	-	-	1.76	1.60-1.94	<0.01	1.26	1.12-1.42	<0.01
Exposure to mass media												
No	-	-	-	-	-	-	1			1		
Yes	-	-	-	-	-	-	0.48	4.24-5.45	<0.01	1.45	1.24-1.70	<0.01
Wealth quintile												
Poorest	1			1			1			1		
Poorer	1.54	1.21-1.95	<0.01	1.49	1.16-1.92	<0.01	1.93	1.59-2.32	<0.01	1.41	1.14-1.74	<0.01
Middle	2.32	1.84-2.94	<0.01	1.94	1.50-2.52	<0.01	2.99	2.49-3.58	<0.01	1.83	1.46-2.28	<0.01
Richer	5.25	4.21-6.56	<0.01	3.15	2.42-4.11	<0.01	6.28	5.27-7.50	<0.01	2.64	2.08-3.36	<0.01
Richest	15.42	12.33-19.28	<0.01	6.07	4.50-8.19	<0.01	20.88	17.33-25.14	<0.01	5.27	3.98-6.98	<0.01
Distance from health facility for medical care												
Big problem	-	-	-	-	-	-	1			1		
Not a big problem	-	-	-	-	-	-	2.76	2.50-3.05	<0.01	0.88	0.74-1.04	0.13
Transport arrangement for medical care												
Big problem	-	-	-	-	-	-	1			1		
Not a big problem	-	-	-	-	-	-	2.92	2.65-3.22	<0.01	1.21	1.03-1.44	0.02

**Table 5:** Bivariate and multivariate logistic regression models of achievement of ANC4+ and SBA (Continued Care at Delivery) with various determinants within each of the two waves of PDHS during 2006-2012

Characteristics	Model II (ANC4+ and SBA)											
	PDHS (2006/07)						PDHS (2012/13)					
	Bivariate			Multivariate			Bivariate			Multivariate		
	OR	CI (95%)	*p*-value	AOR	CI (95%)	*p*-value	OR	CI (95%)	*p*-value	AOR	CI (95%)	*p*-value
Regions/Provinces												
Baluchistan	1			1			1			1		
Punjab	4.66	3.34-6.51	<0.01	3.06	2.10-4.45	<0.01	4.51	3.65-5.58	<0.01	2.65	2.06-3.42	<0.01
Sindh	5.22	3.72-7.33	<0.01	5.49	3.76-8.02	<0.01	6.44	5.19-7.99	<0.01	5.45	4.23-7.01	<0.01
Khyber Pakhtunkhwa	3.09	2.16-4.42	<0.01	2.74	1.84-4.07	<0.01	2.82	2.26-3.53	<0.01	2.29	1.77-2.96	<0.01
Gilgit Baltistan	-	-	-	-	-	<0.01	3.91	3.04-5.01	<0.01	4.46	3.32-6.00	<0.01
Islamabad	-	-	-	-	-	<0.01	25.80	19.46-34.20	<0.01	7.80	5.59-10.89	<0.01
Geographical classification												
Rural	1			1			1			1		
Urban	4.45	3.88-5.10	<0.01	1.55	1.29-1.85	<0.01	3.55	3.21-3.93	<0.01	1.19	1.02-1.37	0.02
Respondents age												
15-24 years	1			1			1			1		
25-34 years	1.24	1.05-1.45	0.01	1.16	0.92-1.45	0.21	1.04	0.93-1.18	0.44	1.04	0.87-1.24	0.67
35 years and above	0.69	0.57-0.85	<0.01	1.08	0.78-1.51	0.64	0.73	0.63-0.85	<0.01	1.09	0.84-1.42	0.51
Respondents age at first birth												
Less than 20 years	1			1			1			1		
20-29 years	1.88	1.64-2.16	<0.01	1.12	0.93-1.35	0.22	2.07	1.86-2.31	<0.01	1.16	1.00-1.35	0.05
30 years and above	2.10	1.49-2.96	<0.01	1.59	0.99-2.54	0.05	4.79	3.70-6.21	<0.01	1.68	1.14-2.47	0.01
Respondents educational status												
Uneducated	1			1			1			1		
Primary	2.89	2.38-3.51	<0.01	1.49	1.20-1.86	<0.01	3.09	2.65-3.61	<0.01	1.42	1.18-1.69	<0.01
Secondary	6.37	5.33-7.62	<0.01	2.08	1.66-2.60	<0.01	7.46	6.49-8.56	<0.01	2.15	1.80-2.58	<0.01
Higher	21.37	16.65-27.43	<0.01	4.62	3.35-6.37	<0.01	20.80	17.39-24.88	<0.01	3.48	2.73-4.44	<0.01
Husbands educational status												
Uneducated	1			1			1			1		
Primary	1.64	1.29-2.09	<0.01	1.03	0.79-1.34	0.81	1.94	1.60-2.35	<0.01	1.10	0.89-1.38	0.36
Secondary	3.44	2.86-4.15	<0.01	1.44	1.16-1.79	<0.01	3.92	3.38-4.54	<0.01	1.45	1.22-1.74	<0.01
Higher	7.65	6.25-9.35	<0.01	1.56	1.20-2.03	<0.01	8.35	7.15-9.74	<0.01	1.43	1.16-1.77	<0.01
Respondents employment status												
Unemployed	1			1			1			1		
Employed	0.56	0.48-0.66	<0.01	0.89	0.73-1.08	0.23	0.58	0.51-0.67	<0.01	0.76	0.64-0.90	<0.01
Husbands employment status												
Unemployed	1			1			1			1		
Employed	1.20	0.82-1.76	0.34	0.97	0.61-1.51	0.88	1.72	1.22-2.41	<0.01	1.24	0.82-1.87	0.31
Parity/Number of children												
5 children or more	1			1			1			1		
3-4 children	1.96	1.64-2.34	<0.01	1.05	0.83-1.31	0.68	2.25	1.96-2.58	<0.01	1.20	0.99-1.45	0.05
1-2 children	2.60	2.20-3.06	<0.01	1.42	1.09-1.85	<0.01	3.81	3.35-4.34	<0.01	1.99	1.59-2.48	<0.01
Respondents autonomy to healthcare decision-making												
No	-	-	-	-	-	-	1			1		
Yes	-	-	-	-	-	-	1.85	1.68-2.05	<0.01	1.30	1.15-1.48	<0.01
Exposure to mass media												
No	-	-	-	-	-	-	1			1		
Yes	-	-	-	-	-	-	5.82	5.04-6.73	<0.01	1.43	1.19-1.71	<0.01
Wealth quintile												
Poorest	1			1			1			1		
Poorer	1.90	1.36-2.66	<0.01	1.72	1.21-2.44	<0.01	2.64	2.10-3.33	<0.01	1.83	1.42-2.36	<0.01
Middle	3.23	2.35-4.44	<0.01	2.40	1.70-3.40	<0.01	4.03	3.23-5.04	<0.01	2.31	1.78-3.01	<0.01
Richer	7.87	5.84-10.61	<0.01	3.86	2.73-5.47	<0.01	9.22	7.44-11.42	<0.01	3.52	2.66-4.66	<0.01
Richest	25.91	19.33-34.73	<0.01	7.57	5.21-10.99	<0.01	31.61	25.42-39.29	<0.01	6.79	4.95-9.31	<0.01
Distance from health facility for medical care												
Big problem	-	-	-	-	-	-	1			1		
Not a big problem	-	-	-	-	-	-	3.21	2.88-3.58	<0.01	0.94	0.78-1.13	0.53
Transport arrangement for medical care												
Big problem	-	-	-	-	-	-	1			1		
Not a big problem	-	-	-	-	-	-	3.35	3.01-3.72	<0.01	1.25	1.04-1.49	0.01

**Table 6:** Bivariate and multivariate logistic regression models of achievement of ANC4+, SBA and PNC (Complete Continued Care) with various determinants within each of the two waves of PDHS during 2006-2012

Characteristics	Model III (ANC4+, SBA and PNC)											
	PDHS (2006-07)						PDHS (2012-13)					
	Bivariate			Multivariate			Bivariate			Multivariate		
	OR	CI (95%)	*p*-value	AOR	CI (95%)	*p*-value	OR	CI (95%)	*p*-value	AOR	CI (95%)	*p*-value
Regions/Provinces												
Baluchistan	1			1			1			1		
Punjab	4.27	2.91-6.26	<0.01	2.60	1.72-3.95	<0.01	4.90	3.91-6.15	<0.01	2.88	2.21-3.75	<0.01
Sindh	5.39	3.66-7.93	<0.01	5.36	3.52-8.15	<0.01	6.46	5.13-8.13	<0.01	5.01	3.85-6.52	<0.01
Khyber Pakhtunkhwa	2.59	1.72-3.92	<0.01	2.20	1.41-3.44	<0.01	2.60	2.04-3.31	<0.01	1.99	1.52-2.62	<0.01
Gilgit Baltistan	-	-	-	-	-	<0.01	1.95	1.46-2.60	<0.01	1.74	1.25-2.43	<0.01
Islamabad	-	-	-	-	-	<0.01	20.72	15.64-27.44	<0.01	5.56	3.99-7.74	<0.01
Geographical classification												
Rural	1			1			1			1		
Urban	4.39	3.76-5.11	<0.01	1.45	1.19-1.76	<0.01	3.35	3.01-3.73	<0.01	1.14	0.98-1.33	0.09
Respondents age												
15-24 years	1			1			1			1		
25-34 years	1.31	1.10-1.57	<0.01	1.18	0.92-1.51	0.18	1.03	0.91-1.17	0.66	0.91	0.76-1.10	0.34
35 years and above	0.67	0.53-0.84	<0.01	1.02	0.71-1.46	0.92	0.76	0.65-0.88	<0.01	1.07	0.82-1.41	0.61
Respondents age at first birth												
Less than 20 years	1			1			1			1		
20-29 years	2.10	1.79-2.46	<0.01	1.28	1.04-1.57	0.02	2.28	2.03-2.55	<0.01	1.26	1.08-1.48	<0.01
30 years and above	2.11	1.43-3.09	<0.01	1.64	0.98-2.72	0.059	5.38	4.15-6.97	<0.01	1.74	1.19-2.56	<0.01
Respondents educational status												
Uneducated	1			1			1			1		
Primary	2.84	2.27-3.54	<0.01	1.46	1.14-1.88	<0.01	3.32	2.81-3.91	<0.01	1.54	1.28-1.87	<0.01
Secondary	5.98	4.91-7.29	<0.01	1.92	1.50-2.46	<0.01	7.01	6.07-8.09	<0.01	2.09	1.73-2.52	<0.01
Higher	17.45	13.69-22.24	<0.01	3.44	2.48-4.77	<0.01	20.05	16.84-23.86	<0.01	3.73	2.92-4.76	<0.01
Husbands educational status												
Uneducated	1			1			1			1		
Primary	1.56	1.18-2.08	<0.01	0.98	0.72-1.33	0.92	2.05	1.67-2.53	<0.01	1.18	0.93-1.49	0.16
Secondary	3.32	2.68-4.12	<0.01	1.42	1.11-1.83	<0.01	4.11	3.50-4.82	<0.01	1.61	1.33-1.95	<0.01
Higher	7.67	6.13-9.60	<0.01	1.60	1.20-2.14	<0.01	8.54	7.24-10.08	<0.01	1.60	1.28-2.00	<0.01
Respondents employment status												
Unemployed	1			1			1			1		
Employed	0.63	0.53-0.75	<0.01	1.03	0.83-1.26	0.81	0.64	0.56-0.74	<0.01	0.81	0.68-0.96	0.02
Husbands employment status												
Unemployed	1			1			1			1		
Employed	1.61	1.01-2.60	0.05	1.29	0.76-2.19	0.35	2.13	1.44-3.16	<0.01	1.38	0.87-2.19	0.16
Parity/Number of children												
5 children or more	1			1			1			1		
3-4 children	2.19	1.78-2.68	<0.01	1.16	0.90-1.49	0.25	2.34	2.02-2.72	<0.01	1.23	1.01-1.50	0.04
1-2 children	2.81	2.32-3.39	<0.01	1.49	1.11-1.99	0.01	3.96	3.45-4.55	<0.01	1.91	1.51-2.39	<0.01
Respondents autonomy to healthcare decision-making												
No	-	-	-	-	-	-	1			1		
Yes	-	-	-	-	-	-	1.77	1.59-1.96	<0.01	1.19	1.04-1.36	0.01
Exposure to mass medias												
No	-	-	-	-	-	-	1			1		
Yes	-	-	-	-	-	-	6.35	5.40-7.48	<0.01	1.48	1.21-1.81	<0.01
Wealth quintile												
Poorest	1			1			1			1		
Poorer	1.62	1.09-2.41	0.02	1.55	1.03-2.34	0.03	2.29	1.76-2.97	<0.01	1.53	1.15-2.03	<0.01
Middle	3.12	2.16-4.50	<0.01	2.51	1.69-3.74	<0.01	3.93	3.07-5.02	<0.01	1.98	1.49-2.64	<0.01
Richer	6.99	4.96-9.85	<0.01	3.73	2.51-5.56	<0.01	8.92	7.05-11.28	<0.01	2.79	2.07-3.78	<0.01
Richest	22.48	16.14-31.32	<0.01	7.38	4.83-11.27	<0.01	31.02	24.55-39.19	<0.01	5.37	3.85-7.49	<0.01
Distance from health facility for medical care												
Big problem	-	-	-	-	-	-	1			1		
Not a big problem	-	-	-	-	-	-	3.30	2.94-3.70	<0.01	1.04	0.85-1.25	0.71
Transport arrangement for medical care												
Big problem	-	-	-	-	-	-	1			1		
Not a big problem	-	-	-	-	-	-	3.36	3.00-3.75	<0.01	1.11	0.92-1.34	0.26

The overall findings highlight an improved trend of seeking care along the pathway of CoC, revealing strong association with several factors. However, a substantial difference in patterns of continued care was observed across some of the determinants, such as amongst the provinces as indicated from Table 3, where respondents from Punjab received more care at all levels as compared to any other province in both waves. For instance, 44 and 30.8% women received complete CoC from Punjab, followed by 38 and 29.2% from Sindh province in waves 2 and 3 respectively. Similarly, the respondents from the richest wealth quintile, preferably of age 25–34 years, living in urban areas, giving a first birth at higher age, having 1–2 children and husbands being employed significantly received more continued care at all levels from both waves, whereas pregnant women being employed themselves were less likely to avail complete CoC. Nevertheless, a significant difference was found regarding respondents education, where mainly uneducated mothers (31.6%) received complete CoC in wave 2, whereas mostly mothers with higher education (31.4%) availed CoC in wave 3. Moreover, CoC was associated with higher healthcare decision making (54.0%), exposure to mass media (91.2%), and having not a big problem in case of distance (76.1%) and transport arrangement to access health facility for medical care (72.9%) in wave 3 of PDHS.

The factors associated with the achievement of ANC4+ are shown in model I (Table 4). Model II is about the achievement of ANC4+ and SBA, which are defined as continued care at delivery (Table 5). Table 6 illustrates the results of model III on complete CoC (ANC4 and SBA and PNC).

Overall, the findings of model I (Table 4) show that women living in urban areas and with higher education were more likely to achieve ANC4+ in PDHS 2006/07 and 2012/13 respectively. Similarly, the odds of ANC4+ was high for the respondents with highly educated husbands (AOR 1.53, 95% CI: 1.21-1.93; AOR 1.23, 95% CI: 1.01-1.49) from waves 2 and 3 respectively. The economic status of respondents showed that the richest respondents had more chances to avail ANC4+ than any other category of economic status within both waves.

Table 5 relates to model II, which is about the achievement of ANC4+ and SBA (continued care at delivery). Most importantly, results depict that respondents from Sindh and living in urban areas were more likely to achieve ANC4+ and SBA in waves 2 and 3 respectively. A high odds ratio was found for the association between achievement of ANC4+ and SBA and a high educational status (AOR 4.62, 95% CI: 3.35-6.36; AOR 3.48, 95% CI: 2.73-4.44) in both waves. Moreover, respondents whose husbands received higher education (AOR 1.56, 95% CI: 1.20-2.03; AOR 1.43, 95% CI: 1.16-1.77), who have only 1–2 children (AOR 1.42, 95% CI: 1.09-1.85; AOR 1.99, 95% CI: 1.59-2.48) and belong to the richest wealth quintile (AOR 7.57, 95% CI: 5.21-10.99; AOR 6.79, 95% CI: 4.95-9.31) had a higher likelihood of continued care at delivery (ANC4+ and SBA) than any other category during wave 2 and 3 respectively.

Table 6 illustrates the results of model III, complete CoC (ANC4 and SBA and PNC), which highlights that the respondents from Sindh from wave 2 and 3, as well as from Islamabad from wave 3 were more likely to have complete CoC. The result for females who gave birth at the age of 30 years age or above were insignificant for the second wave of PDHS (AOR 1.64, 95% CI: 0.98-2.72) but significant for the third wave (AOR 1.74, 95% CI: 1.19-2.56). Respondents with higher educational status (AOR 3.44; 95% CI: 2.48-4.77; AOR 3.73, 95% CI: 2.92-4.76), having 1–2 children (AOR 1.49, 95% CI: 1.11-1.99; AOR 1.91, 95% CI: 1.51-2.39) and belonging to the richest quintile (AOR 7.38; 95% CI: 4.83-11.27; AOR 5.37, 95% CI: 3.85-7.49) were more likely to achieve complete CoC from wave 2 and 3 accordingly. Moreover, the odds of respondents autonomy to healthcare decision making (AOR 1.19, 95% CI: 1.04-1.36) and their exposure to the mass media (AOR 1.48, 95% CI: 1.21-1.81) was also found higher to achieve complete CoC in PDHS 2012/13.

## Discussion

The continuum of care has become a core strategy for reducing maternal, newborn and child mortality. CoC promotes integrated MNCH services, connecting three components of maternal care (ANC, SBA and PNC). Women’s childbearing years, infancy and childhood are widely known as critical junctures for lifelong health and productive populations, which is influenced by various key factors. Improving MNCH services is the explicit focus of each country including Pakistan to better understand the underlying gaps in seeking care along the pathway of continuum of care [21]. Thus, we analysed the perspective of the continuum of care to identify factors that affect women’s continuation in receiving care from pregnancy to childbirth and after delivery.

Overall, CoC completion rate has increased over time, which might be attributable to older ages at first birth and higher level of education in 2012 compared to 2006. Nevertheless, women in Pakistan still lack continuum of care at all three levels. The greatest gap and contributor to low CoC was detected at the initial stage, i.e. ANC followed by SBA and PNC. The main reason is the non-achievement of the optimal number of ANC visits coupled with home based deliveries, where more than half of deliveries are conducted at home through traditional birth without any skilled assistance [22]. However, the trends of availing PNC services has somehow increased, nonetheless, altogether continuum of care was seen very low in Pakistan. The fact behind the failure of seeking complete CoC is that after receiving ANC, a majority of the women dropped out from the pathway of continued care and did not receive skilled birth assistance or PNC for themselves or for their newborns. Findings from both waves of PDHS suggest that more dropouts occurred between pregnancy and delivery than between delivery and PNC period, which is similar to studies conducted in Asia or Africa [13, 23–25].

Receiving antenatal care is considered a significant predictor of subsequent use of skilled assistance during delivery. High quality ANC visits make women better informed about pregnancy and more likely to recognize the importance of SBA. Most women who had skilled assistance at delivery also continued to receive PNC from professional and skilled healthcare providers during the first 48 h, in case they delivered at a health facility. These findings demonstrate that increasing the use of SBA, especially conducting delivery at health facilities could lead to more use of PNC and thus improve the continuity of care in Pakistan. A recent multi-country analysis of Africa had similar findings where ANC has a positive effect on delivery assistance and PNC [26].

We found that a majority of the determinants related to respondents reproductive status, such as higher age at first birth and less number of children, coupled with the community context including developed regional location and urban areas are strongly associated with utilization of CoC and its components (ANC, SBA and PNC) in Pakistan. Likewise, within the family influences, husbands’ higher education, high wealth status and exposure to mass media, as well as respondents’ higher education and autonomy from social and cultural factors influenced CoC completion rate. These findings are similar to other research conducted in Ghana, Tanzania, India and China [27–29]. Particularly, the role of women autonomy and exposure to mass media in determining maternal health utilization is evident from various research [30–34]. Whereas unemployment was described as a predictor of late initiation for prenatal care [35], our study shows contradictory findings. The higher likelihood of not completing CoC in employed women might be due to the fact that employed women have time barriers to access services.

Summarizing above, the reasons of discontinued CoC may include but are not limited to traditional cultural practices, lack of health education and awareness raising sessions, non-availability and non-affordability of services, shortage of trained/competent female staff, deficient drugs and equipment and the weaker performance of our health system.

### Limitations

Since this research involved two waves of the PDHS, some limitations were faced during data analysis, e.g. lack of uniformity in variables including respondent’s autonomy, exposure to mass media, distance to health facility and transport arrangement for medical care. Likewise, no data about PNC variables within the first wave of PDHS 1990/91 were available. Therefore, the analysis was limited to the two latest waves of PDHS from 2006 to 2012.

Further limitations apply to the analysis, which are due to the design of PDHS. Firstly, no causal relationships can be displayed because of the cross-sectional nature of the survey. Secondly, information in the survey is based on self-reports and may be, therefore, biased.

## Conclusion

This research presented the trends of a composite measure of CoC from the pregnancy to postpartum stages, highlighting that complete COC has increased from 15% to 27% amongst respondents over time from 2006 to 2012. The greatest gap and contributor to discontinued care was observed at pregnancy level, in achieving ANC4+ followed by SBA at delivery level and PNC within 48 h during postpartum period. The factors associated with low CoC completion rate include various determinants of reproductive status, family influence, community context and social and cultural beliefs.

The study findings call for attention to practical interventions targeted at enhancing ANC for women, thereby increasing CoC completion rate. Quality of ANC is connected to women’s use of SBA and PNC and should be emphasized. Such interventions should be tailored focusing on the needs of women in Pakistan to provide CoC in an integrated manner involving both public and private sector to strengthen health systems for appropriately addressing the factors hindering CoC completion. Future program efforts should focus on advocacy, ensuring the availability of skilled workers, community mobilizations and quality services through engaging key stakeholders and development partners for promoting CoC in Pakistan.

## Abbreviations

ANC: Antenatal care
ANC4+: Four or more antenatal care visits
AOR: Adjusted odds ratio
CoC: Continuum of Care
CI: Confidence interval
LHV: Lady Health Visitor
LMIC: Low and Middle Income Countries
MDG: Millennium Development Goals
MNCH: Maternal, Newborn and Child Health
OR: Odds ratio
PDHS: Pakistan Demographic and Health Survey
PNC: Postnatal care
SBA: Skilled Birth Attendance
SDG: Sustainable Development Goals
SPSS: Statistical Package of Social Science
USAID: US Agency for International Development
WHO: World Health Organization
